# Co-creation of injury prevention measures for competitive adolescent distance runners: knowledge, behavior, and needs of athletes and coaches enrolled on England Athletics’ Youth Talent Programme

**DOI:** 10.1080/07853890.2024.2334907

**Published:** 2024-04-10

**Authors:** Robert H. Mann, Bryan C. Clift, Jo Day, Alan R. Barker

**Affiliations:** a Children’s Health and Exercise Research Centre, Department of Public Health and Sport Sciences, Faculty of Health and Life Sciences, University of Exeter, Exeter, UK; b Department for Health, University of Bath, Bath, UK; c Centre for Qualitative Research, University of Bath, Bath, UK; d NIHR Applied Research Collaboration South West Peninsula, University of Exeter, Exeter, UK

**Keywords:** Athletics, track and field, youth sport, athlete health protection, co-production

## Abstract

This study assessed the knowledge, behavior, and needs of competitive adolescent (16–18 years) distance runners and distance running coaches enrolled as part of England Athletics’ Youth Talent Programme in relation to the prevention of running-related injury (RRI). Two online surveys were developed and distributed to the distance runners (survey one) and coaches (survey two). Both surveys included sections related to: (1) current knowledge; (2) current behavior; (3) need and support for RRI prevention measures; and (4) possible content and form of RRI prevention measures. A total of 39 distance runners (36% of total possible sample) completed survey 1, and 29 coaches (32% of total possible sample) completed survey 2. Key findings included that the majority of distance runners and coaches: (1) agreed that it is ‘very important’ to try to prevent RRI; (2) are currently implementing something in practice (e.g., strength training) to prevent RRI; and (3) view the creation of RRI prevention measures as an important initiative. Differences between distance runners and coaches were identified in relation to their understanding of the most common causes of RRI. Interestingly, distance runners identified a modifiable cause of RRI (i.e., too much training) as the most common cause of RRI, while coaches selected a non-modifiable cause of RRI (i.e., growth and maturation). These key findings were supplemented by competitive adolescent runners and distance running coaches detailing their delivery preferences for such RRI prevention measures. Results from this study will help inform subsequent steps of the larger co-creation process, with an emphasis on developing multifaceted and context-specific RRI prevention measures that are deemed to be feasible and acceptable for real-world implementation.

## KEY MESSAGES

- The majority of competitive adolescent distance runners and distance running coaches who took part in this study agreed that it is ‘very important’ to try to prevent RRI and view the creation of RRI prevention measures as an important initiative.
- Alongside this support, interesting differences between the distance runners and coaches were also identified, including, for example: (1) their understanding of the most common causes of RRI and (2) their preferences about where and when to complete RRI prevention measures.
- Results from this study will help inform subsequent steps of the larger co-creation process, with an emphasis on developing multifaceted and context-specific RRI prevention measures that are deemed to be feasible and acceptable for real-world implementation.

## Introduction

Distance running is a popular sport among adolescents around the world [1]. Based on Sport England’s Active Lives Survey data [2,3], distance running is often ranked as the second most participated in activity among children and young people (5–16 years) and adults (16+ years). The popularity of distance running is also illustrated by the number of adolescents who take part competitively, often as part of Athletics (Track and Field). Despite participation in distance running being associated with a number of health benefits in later life [4], research highlights that participation can also lead to negative health outcomes, such as running-related injury (RRI) [5–9]. In adolescent distance runners, the incidence of RRI ranges from 0.84 to 25.0 per 1000 h of participation [10–15], and the prevalence of RRI ranges from 15% to 32% [15–18]. The lower limb is the most commonly injured body region for adolescent distance runners [13–15, 18,19], while RRI is often reported to have a ‘gradual onset’ (related to overuse) [8, 20]. Although RRI can adversely influence an adolescent distance runner’s training availability and/or performance, RRI is also likely to affect their overall psychosocial wellbeing [21,22]. This is especially the case when being ‘competitive’ (i.e., performance focused) is part of an adolescent’s identity [23]. As a consequence, multifaceted and context-specific preventative measures need to be created in order to effectively reduce the risk of RRI and protect the health of adolescent distance runners.

Despite a range of adult-based injury prevention measures having been created (and implemented) for distance runners [24–27], and Athletics athletes [28], there is a lack of such measures for adolescent distance runners [5, 29]. To our best knowledge, the only relevant example is a universal prevention digital health platform designed for 12–15-year-old Swedish Athletics athletes (all event groups) [30,31]. Therefore, in partnership with England Athletics, co-creating RRI prevention measures for competitive adolescent distance runners enrolled on their Youth Talent Programme (YTP) was identified as a priority. The YTP represents the first step on England Athletics’ talent pathway and is designed to meet the needs of identified, talented 16–18-year-old English athletes (all event groups) and their coaches – largely focused on developing an athlete’s dual-career (i.e., combining elite sport and academic/employment opportunities) [32].

To increase the likelihood of effective real-world implementation of such injury prevention measures, this work foregrounded the value of integrating key stakeholders throughout the co-creation process to develop multifaceted and context-specific preventative measures for the YTP [33]. This is a necessary and increasingly popular approach in the sport, exercise, and health sciences [34]. Specific to this project, an iterative six-stage co-creation process was undertaken, including input from the following stakeholder groups: (1) competitive adolescent distance runners; (2) parents/carers of competitive adolescent distance runners; (3) distance running coaches; (4) healthcare practitioners; and (5) research scientists. As the second stage of this larger co-creation process, the purpose of this study was to assess the knowledge, behavior, and needs of competitive adolescent distance runners and distance running coaches enrolled as part of England Athletics’ YTP in relation to RRI prevention.

## Methods

### Study design

This was a cross-sectional study based on the completion of two online surveys. These surveys were designed for and distributed to: (1) distance runners enrolled on England Athletics’ YTP and (2) their affiliated distance running coaches. Data collection took place between 1 March 2021 and 30 April 2021. Access to both surveys was provided via Qualtrics XM (Seattle, WA). The results from this study were used to inform a series of workshops as part of a larger project aimed at co-creating RRI prevention measures for competitive adolescent distance runners.

### Participants

All distance runners enrolled on England Athletics’ YTP (*n* = 109) at the time of data collection, and their affiliated distance running coaches (*n* = 90), were invited to participate in this study. Beyond being enrolled on England Athletics’ YTP, inclusion criteria for the distance runners included: (1) being aged between 16 and 18 years old; (2) being based in England; (3) currently training for and/or competing in distance running events from 800 m up to 10,000 m, including steeplechase; and (4) being able to speak and understand a sufficient level of English. Inclusion criteria for distance running coaches included: (1) having an up-to-date England Athletics coaching accreditation; (2) being a coach of a distance runner who was enrolled on England Athletics’ YTP; and (3) being able to speak and understand a sufficient level of English.

The distance runners who were invited to participate in this study are defined as ‘competitive’ as they align to a semi-elite classification [35], while also spanning Tier 2 (i.e., trained/developmental) and Tier 3 (i.e., highly trained/national level) of the Participant Classification Framework [36]. These runners were also defined as ‘adolescent’ as England Athletics’ YTP is specifically for athletes aged between 16 and 18 years old – best aligned to the term ‘older adolescents’ (15–19 years) [37]. Although it is harder to define the distance running coaches invited to participate in this study, they can broadly be viewed as ‘youth endurance event group coaches.’ This is justified by the fact that they currently coach at least one distance runner enrolled on England Athletics’ YTP, while holding relevant coaching qualifications.

Eligible distance runners and distance running coaches were separately sent (via email) information about how to participate in this study (i.e., a participant information sheet and survey weblink) by England Athletics’ YTP Lead. The same England Athletics’ member of staff sent out reminder emails to all potential participants who had not completed the survey after 21 days of non-response. If no response followed, these potential participants were categorized as ‘non-responders.’

### Ethics approval

As part of the larger co-creation project, ethics approval was granted by the University of Exeter’s College of Medicine and Health Research Ethics Committee (Approval Reference: 20/12/271) for this study. All participants provided consent to participate by completing the electronic consent form at the start of each online survey. Before completing the consent form, participants were provided with a participant information sheet which outlined the aims, methods, and associated risk/benefits of taking part in the study. Participants were also provided with the contact details of the research team, should they wish to discuss their participation, prior to providing consent. Due to being aged between 16 and 18 years old, obtaining parental consent was not required for the adolescent distance runners taking part in this study. However, as part of their information sheet, it was recommended that the participants discussed their participation in this study with their parents/carers. Participants were free to withdraw from the study at any time before data analysis was undertaken, as also detailed in the relevant information sheets.

### Surveys

All research team members supported the design of two online surveys. These surveys aimed to assess the current knowledge and behavior of distance runners enrolled on England Athletics’ YTP (survey one), and their affiliated coaches (survey two), in relation to RRI prevention. Both surveys also explored the perceived need and support for creating RRI prevention measures, including questions about the possible content and form of such measures. Each survey included the following six sections: (1) background information; (2) current knowledge; (3) current behavior; (4) need and support for RRI prevention measures; (5) content and form of RRI prevention measures; and (6) future project involvement. A short introduction and an electronic consent form were included at the start of each survey.

Survey one (see Supplementary File 1) included a total of 30 mandatory questions, while survey two (see Supplementary File 2) included a total of 25 mandatory questions. Optional follow-up questions were used in both surveys to enable participants to give more details about certain question responses. These optional follow-up questions were presented according to prior responses, as part of the survey flow. Both surveys included a combination of dichotomous, multiple-choice, Likert scale, matrix table, and open text entry question types. Responses to the open text entry questions are not reported in this article, due to predominantly being non-mandatory questions. Responses to these questions were used to shape discussions in a series of workshops as part of the larger project. Full lists of survey questions are detailed in Supplementary Files 1 and 2.

### Patient and public involvement

The surveys were developed with input from two adolescent distance runners, two qualified athletics coaches, and two England Athletics’ staff members. This feedback was related to the appropriateness of the content, look, and flow of these surveys. Where possible, suggested changes were incorporated into the final survey designs. For example, having a follow-up question when participants selected ‘other’ as a response was hidden from view (via survey logic) until this selection was made to improve user experience.

### Statistical analysis

Prior to data analysis, data from both surveys were coded and combined in Microsoft Excel (version 2306; Microsoft, Redmond, WA). This combined dataset was then transferred to IBM SPSS Statistics (version 28.0; IBM, Armonk, NY) for analysis. Categorical variables and survey items that had Likert scale items were presented as frequencies and percentages. For these variables, Chi-square test of independence (*χ*^2^) was performed to determine if there were differences in frequencies and percentages between two groups. Statistical significance was set at an alpha level of .05.

## Results

### Participant characteristics

Overall, fully complete (i.e., answered all questions) surveys were received from 44 distance runners and 31 coaches. One incomplete survey was submitted by a coach and was removed prior to analysis. Of the fully complete surveys, a total of seven survey responses were removed prior to analysis for the following reasons: (1) a distance runner (*n* = 1) and coach (*n* = 1) were not enrolled on England Athletics’ YTP and (2) distance runners (*n* = 4) and a coach (*n* = 1) completing the survey twice. For the latter reason, each participant’s second survey submission was removed prior to analysis.

Accounting for the removed responses, a total of 39 distance runners (24 female) completed survey one, representing 36% of the total possible sample. A total of 29 (six female) coaches completed survey two, representing 32% of the total possible sample. The median survey completion time for all participants was 20 min. Participant characteristics for both the distance runners and coaches are provided in Table 1. Preliminary analyses revealed no sex differences in the observed responses, so data are combined for males and females for subsequent analyses.

**Table 1.** Characteristics of competitive adolescent distance runners and distance running coaches.

Characteristics	Distance runners (*n* = 39)	Distance running coaches (*n* = 29)
Sex		
Female	24 (62%)	6 (21%)
Male	15 (38%)	23 (79%)
Chronological age, years (SD)	17.1 (0.5)	–
Training age, years (SD)	3.4 (1.1)	–
YTP cohort		
2019/2021	7 (18%)	5 (17%)
2020/2022	32 (82%)	23 (79%)
Both	–	1 (3%)
Main event		
800 m	10 (27%)	–
1500 m	11 (28%)	–
3000 m	11 (28%)	–
5000 m	4 (10%)	–
Steeplechase	3 (8%)	–
Level of sport specialization^a^		
High	24 (62%)	–
Moderate	13 (33%)	–
Low	2 (5%)	–
Years of coaching experience		
1–5 years	–	6 (21%)
6–10 years	–	12 (41%)
11–15 years	–	5 (17%)
>15 years	–	6 (21%)
Number of athletes in training group		
1–10 athletes	–	10 (35%)
11–20 athletes	–	10 (35%)
>20 athletes	–	9 (31%)
Number of coached training sessions per week^b^		
1–2 sessions	–	8 (27%)
3–4 sessions	–	19 (66%)
>4 sessions	–	2 (7%)

*n*: number; SD: standard deviation; YTP: Youth Talent Programme.

Data are presented as *n* and %, unless otherwise stated. Due to rounding, not all numbers add up to stated *n*.

^a^ Level of sport specialization was calculated according to participant responses to three binary (yes/no) questions related to: (1) whether distance running was their main sport; (2) if they had quit other sports to focus on distance running; and (3) if they train/participate in distance running for more than 8 months a year – aligned to work of Jayanthi et al..

^b^ Response was related to the number of coached training sessions per week before COVID-19 pandemic.

### Current knowledge

Participant responses to survey questions about current knowledge, in relation to RRI prevention, are shown in Table 2. There was a significant difference in the proportion of distance runners and coaches for how big the risk of sustaining an RRI is perceived to be. A higher proportion of coaches compared to distance runners believe that the risk of sustaining an RRI is ‘high,’ while the reverse is true for believing that the risk of sustaining an RRI is ‘low’ or ‘moderate.’ There was also a significant difference in the proportion of distance runners and coaches for how they would rate their current knowledge about RRI prevention. A higher proportion of coaches compared to distance runners view their current knowledge as ‘excellent’ or ‘good,’ while the reverse is true when viewing their current knowledge as ‘average’ or ‘poor.’ There was no difference in the proportion of distance runners or coaches in relation to how important they currently think it is to try to prevent RRI. The majority of distance runners and coaches viewed trying to prevent RRI as ‘very important.’

**Table 2.** Questions and responses about the current knowledge and behavior of distance runners and coaches in relation to RRI prevention.

Survey question	All (*n* = 68)	Distance runners (*n* = 39)	Coaches (*n* = 29)	Chi-square
*How big is the risk of sustaining a RRI to adolescent distance runners?* [K]				0.003
High	20 (29%)	6 (15%)	14 (48%)	
Moderate	42 (62%)	27 (69%)	15 (52%)	
Low	6 (9%)	6 (15%)	0 (0%)	
*How important do you think it is to try to prevent RRI?* [K]				0.304
Very important	61 (88%)	34 (87%)	27 (93%)	
Important	4 (6%)	2 (5%)	2 (7%)	
Moderately important	3 (4%)	3 (8%)	0 (0%)	
Somewhat important	0 (0%)	0 (0%)	0 (0%)	
Not important	0 (0%)	0 (0%)	0 (0%)	
*How would you rate your current knowledge about RRI prevention?* [K]				0.046
Excellent	6 (9%)	1 (3%)	5 (17%)	
Good	38 (56%)	20 (51%)	18 (62%)	
Average	22 (32%)	16 (41%)	6 (21%)	
Poor	2 (3%)	2 (5%)	0 (0%)	
Very poor	0 (0%)	0 (0%)	0 (0%)	
*In a normal week, do you currently do anything to try and prevent RRI?* [B]				0.427
Yes	61 (90%)	34 (87%)	27 (93%)	
No	0 (0%)	0 (0%)	0 (0%)	
Sometimes	7 (10%)	5 (13%)	2 (7%)	
Don’t know	0 (0%)	0 (0%)	0 (0%)	
*Have you previously received advice about implementing RRI prevention measures?* [B]				0.001
Yes	51 (75%)	35 (90%)	16 (55%)	
No	17 (25%)	4 (10%)	13 (45%)	
Don’t know	0 (0%)	0 (0%)	0 (0%)	

*n*: number; RRI: running-related injury; [K]: a question related to current knowledge; [B]: a question related to current behavior.

Data are presented as *n* and %. Due to rounding, not all numbers add up to stated *N*. The location of any observed differences is explained in ‘Results’ section.

**Figure 1.**
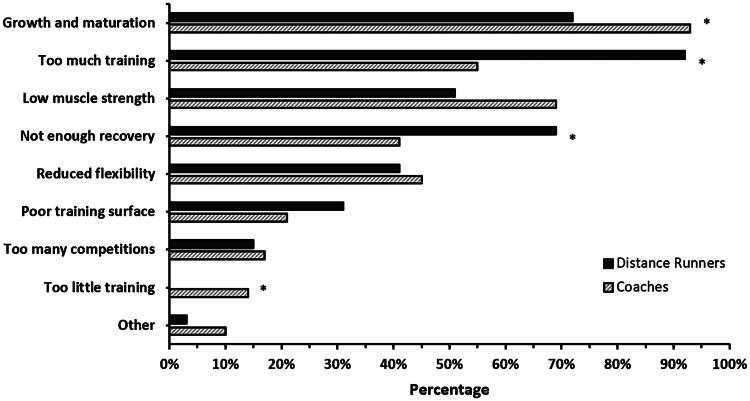
Current understanding of the most common causes of running-related injury for distance runners and coaches. The different causes of running-related injury are shown in order from most (top) to least (bottom) commonly selected by all participants. ‘Other’ causes of running-related injury included: (1) inconsistent training, (2) inappropriate footwear, (3) muscle imbalances, and (4) poor nutrition. Please note that all percentages were rounded. *Chi-squared <0.05.

Participants’ understanding of the most common causes (i.e., risk factors) of RRI is represented in Figure 1. A greater proportion of coaches compared to athletes viewed growth and maturation (*χ*^2^ = 0.027) and too little training (*χ*^2^ = 0.017) as the most common cause of RRI. Conversely, a greater proportion of distance runners compared to coaches viewed too much training (*χ*^2^ < 0.001) and not enough recovery (*χ*^2^ = 0.022) as the most common cause of RRI. This resulted in group differences for the three most common causes of RRI. For distance runners: (1) too much training; (2) growth and maturation; and (3) not enough recovery, and for coaches: (1) growth and maturation; (2) low muscle strength; and (3) too much training.

**Figure 2.**
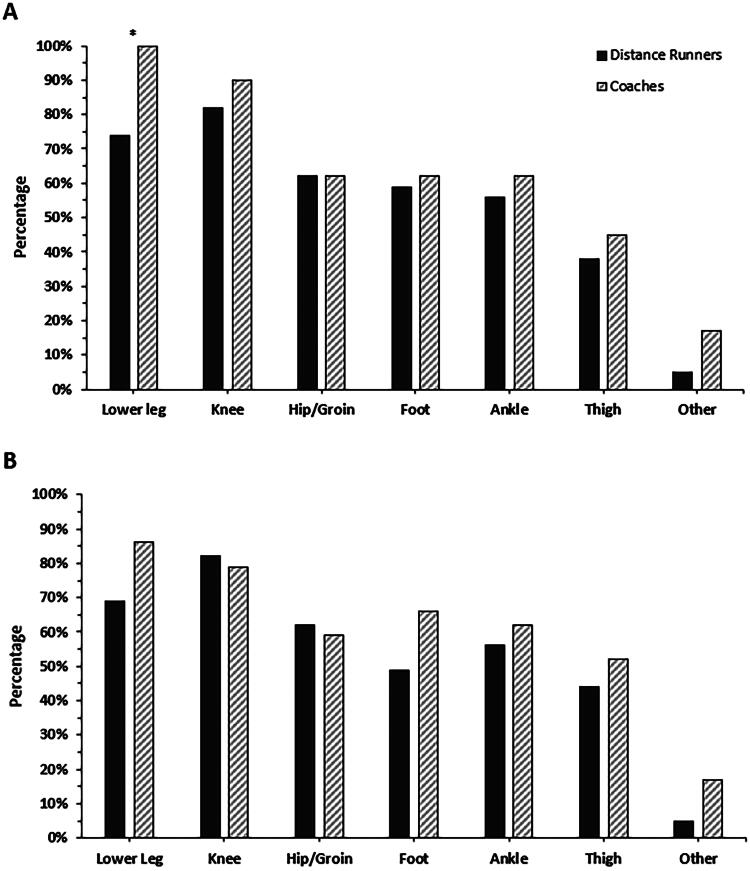
Types of running-related injuries that distance runners and coaches currently know adolescent distance runners are exposed to (A) and believe are important to prevent (B). In (A), types of running-related injuries are shown in order from ‘most’ (left) to ‘least’ (right) commonly selected by all participants. To enable comparison, this ordering is maintained in (B). ‘Other’ types of running-related injuries included: (1) lower back, (2) spine, (3) bone stress, and (4) all injuries. Note that all percentages were rounded. *Chi-squared <0.05.

Figure 2 illustrates the types of RRI that distance runners and coaches know that adolescent distance runners are exposed to and believe are important to prevent. There was general alignment between responses within groups. For 11 of the 14 options, the proportion of coaches who selected the different types of RRI distance runners were exposed to and believed were important to prevent was higher than that reported by the distance runners. A greater proportion of coaches compared to distance runners identified the lower leg as an RRI that adolescent distance runners are exposed to and important to prevent (*χ*^2^ = 0.003).

### Current behavior

Participant responses to survey questions about current behavior, in relation to RRI prevention, are shown in Table 2. There was a significant difference in the proportion of distance runners and coaches for whether they had previously received advice about implementing RRI prevention measures. A higher proportion of distance runners compared to coaches have previously received such advice. There was no difference in the proportion of distance runners or coaches in relation to whether they currently do anything to prevent RRI in a normal training week. Notably, all distance runners and coaches stated that they either ‘did’ or ‘sometimes did’ (as multiple-choice response options) things to prevent RRI in a normal training week.

Table 3 includes details about the type of RRI prevention measures implemented by distance runners and coaches, including how many times these are completed per week. For distance runners, the top three most commonly implemented RRI prevention measures were: (1) use of a warm-up; (2) use of a cool-down; and (3) strength training. For coaches, the top three most commonly implemented RRI prevention measures were: (1) use of a warm-up; (2) running-specific drills; and (3) use of a cool-down. For distance runners and coaches, use of a specific prevention protocol was the least commonly implemented type of RRI prevention measure.

**Table 3.** Types of RRI prevention measures implemented by distance runners and coaches, including weekly frequency.

	Athletes (*n* = 39)			Coaches (*n* = 29)		
Type of RRI prevention measures	None	1–2 times per week	>2 times per week	None	1–2 times per week	>2 times per week
Use of warm-up	3 (8%)	2 (5%)	34 (87%)	1 (3%)	8 (28%)	20 (71%)
Use of cool-down	3 (8%)	2 (5%)	34 (87%)	3 (10%)	7 (24%)	19 (66%)
Balance and coordination training	14 (36%)	20 (51%)	5 (13%)	6 (21%)	16 (55%)	7 (24%)
Flexibility training	12 (31%)	16 (41%)	11 (28%)	5 (17%)	18 (62%)	6 (20%)
Strength training	3 (8%)	22 (56%)	14 (36%)	6 (21%)	21 (72%)	2 (7%)
Circuit training	7 (18%)	19 (49%)	13 (33%)	5 (17%)	22 (76%)	2 (7%)
Stretching pre-run	7 (18%)	3 (8%)	29 (75%)	14 (48%)	6 (21%)	9 (31%)
Stretching post-run	9 (23%)	2 (5%)	28 (73%)	9 (31%)	5 (17%)	15 (52%)
Running-specific drills	4 (10%)	8 (21%)	27 (69%)	2 (7%)	13 (45%)	14 (48%)
Use of a specific prevention protocol	28 (72%)	6 (15%)	5 (13%)	17 (59%)	7 (24%)	5 (17%)

*n*: number; RRI: running-related injury.

Data are presented as *n* and %. Due to rounding, not all numbers add up to stated *N*.

### Need and support

**Figure 3.**
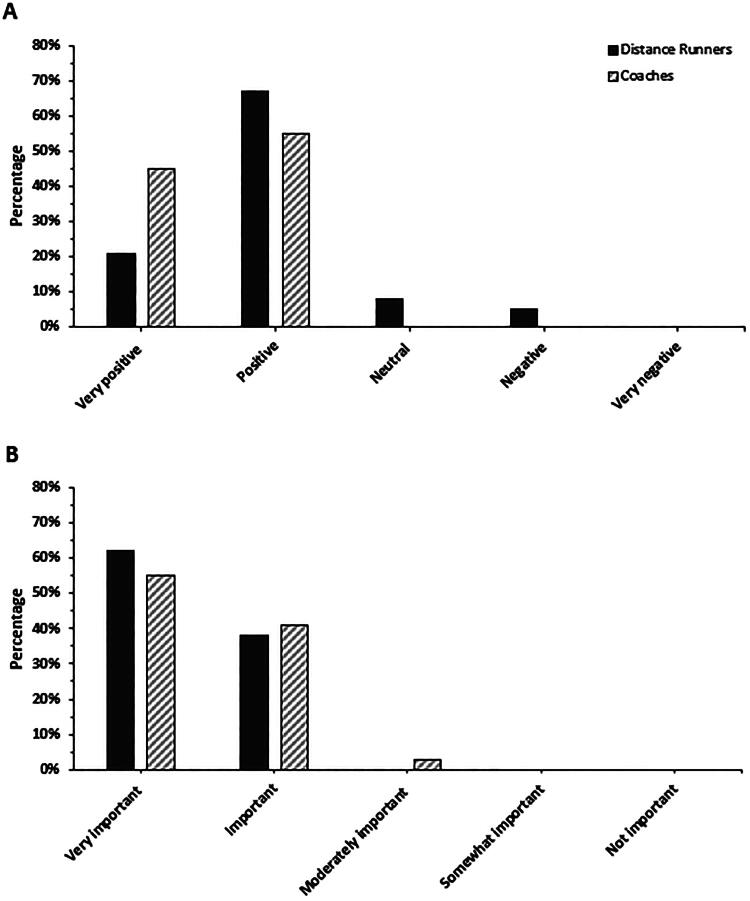
How distance runners and coaches currently feel about injury prevention measures (A) and think about if developing context-specific injury prevention measures is an important initiative (B). Both (A) and (B) are shown in order from ‘most’ (left) to ‘least’ (right) supportive Likert scale response. Note that all percentages were rounded.

Figure 3 presents how distance runners and coaches currently feel about injury prevention measures, in general, and whether they think that developing RRI prevention measures is an important initiative. These data indicate that 88% of distance runners (*n* = 34) and 90% of coaches (*n* = 29) feel ‘very positive’ or ‘positive’ about injury prevention measures, with no differences between groups (*χ*^2^ = 0.064). In relation to whether the development of context-specific RRI prevention measures is an important initiative, 100% of distance runners (*n* = 39) and 96% of coaches (*n* = 29) stated that such an initiative was ‘very important’ or ‘important,’ with no group differences (*χ*^2^ = 0.474).

In response to the question ‘If injury prevention measures were developed, would you adopt them?’, 77% of distance runners (*n* = 30) and 72% of coaches (*n* = 21) agreed that they would adopt them. The remaining participants indicated that they did not know whether they would adopt these measures, rather than disagreeing with the question (i.e., responding ‘no’). There was no difference (*χ*^2^ = 0.671) in the proportion of distance runners or coaches in relation to whether they would adopt the injury prevention measures.

### Possible content and form

Participant responses to survey questions about their delivery preferences for RRI prevention measures are provided in Table 4. In relation to how participants would like to receive information about RRI prevention measures, there were significant differences in the proportion of distance runners and coaches who would like to receive information via a ‘qualified athletics coach’ or ‘workshop/seminar series.’ A higher proportion of coaches would like to receive information via both of these options. There were no other differences in the proportion of distance runners or coaches in relation to how they would like to receive information about RRI prevention measures.

**Table 4.** Questions and responses from distance runners and coaches about their delivery preferences for RRI prevention measures.

Survey question	All (*n* = 68)	Distance runners (*n* = 39)	Coaches (*n* = 29)	Chi-squared
*How would you/your athletes like to receive information about RRI prevention measures?* ^a^				
Via a qualified athletics coach	45 (66%)	22 (56%)	23 (79%)	0.048
Via email	30 (44%)	20 (51%)	10 (35%)	0.168
Via England Athletics’ website	29 (43%)	17 (44%)	12 (41%)	0.885
Via a workshop/seminar series	27 (40%)	11 (28%)	16 (55%)	0.025
Via tailored video content	26 (38%)	13 (33%)	13 (45%)	0.335
Via specific (tailored) website	23 (34%)	11 (28%)	12 (41%)	0.256
Via tailored social media posts	22 (32%)	10 (26%)	12 (41%)	0.170
Via a smart phone application	22 (32%)	12 (31%)	10 (35%)	0.105
Via own athletics club website	12 (18%)	4 (10%)	8 (28%)	0.064
Via a series of infographics	10 (15%)	5 (13%)	5 (17%)	0.611
No preference	6 (9%)	3 (8%)	3 (10%)	0.703
*Who would you/your athletes like to be delivered information about RRI prevention measures from?* ^a^				
Strength and conditioning coach	49 (72%)	25 (64%)	24 (83%)	0.090
Athletics coach	42 (62%)	21 (54%)	21 (72%)	0.119
Physiotherapist/physician	41 (60%)	22 (56%)	19 (66%)	0.448
Professional athlete	32 (47%)	16 (41%)	16 (55%)	0.248
No preference	11 (16%)	9 (23%)	2 (7%)	0.073
*Where would you/your athletes like to complete RRI prevention measures?*				<0.001
At home	37 (54%)	29 (74%)	8 (28%)	
At local athletics club	22 (32%)	10 (26%)	12 (41%)	
At school, academy, or college	0 (0%)	0 (0%)	0 (0%)	
Other^b^	1 (2%)	0 (0%)	1 (3%)	
Don’t know	8 (12%)	0 (0%)	8 (28%)	
*When would you/your athletes like to include RRI prevention measures into a training schedule?*				0.041
As part of a training session	30 (44%)	14 (36%)	16 (55%)	
At a different time to training	35 (52%)	25 (64%)	10 (35%)	
Other^c^	2 (3%)	0 (0%)	2 (7%)	
Don’t know	1 (2%)	0 (0%)	1 (3%)	

*n*: number; RRI: running-related injury.

Data are presented as *n* and %. Due to rounding, not all numbers add up to stated *N*. The location of any observed differences is explained in ‘Results’ section.

^a^ Participants were able to respond ‘yes’ or ‘no’ (binary choice) to each of the options presented as part of these survey questions. Options are listed according to overall (i.e., ‘All’) preference.

^b^ Other included ‘at the gym.’

^c^ Other included a combination of ‘as part of a running training session’ and ‘at a different time to training.’

There were no differences in the proportion of distance runners or coaches in terms of who they would like to be delivered information about RRI prevention measures from. However, there were significant differences in the proportion of distance runners and coaches in relation to: (1) where they would like to complete RRI prevention measures and (2) when they would like to include such measures into a training schedule. These data suggest that: (1) a higher proportion of distance runners would prefer to complete RRI prevention measures ‘at home,’ while coaches favored completion ‘at the local athletics club,’ and (2) a higher proportion of coaches would prefer to integrate RRI prevention measures ‘as part of a running training session,’ while distance runners favored completing such measures ‘at a different time to training.’

## Discussion

This study provides a unique and detailed insight about the knowledge, behavior, and needs of competitive adolescent distance runners enrolled on England Athletics’ YTP, and affiliated distance running coaches, in relation to RRI prevention. Key findings included that the majority of distance runners and coaches: (1) agreed that it is ‘very important’ to try to prevent RRI; (2) are currently implementing something in practice (e.g., strength training) to prevent RRI; and (3) view the creation of RRI prevention measures as an important initiative. Notably, interesting differences between distance runners and coaches were identified in relation to their understanding of the most common causes of RRI. These key findings were supplemented by competitive adolescent runners and distance running coaches detailing their delivery preferences for such RRI prevention measures – aiding subsequent steps of the larger co-creation process.

### Current knowledge

More than 90% of all participants indicated that the risk of sustaining RRI was either ‘moderate’ or ‘high.’ This aligns with current available evidence, whereby a large number of RRIs are sustained by adolescent distance runners and it is acknowledged that distance running regularly accounts for a large proportion of Athletics-related injuries [5, 12, 14, 19]. The fact that a higher proportion of coaches compared to distance runners believed that the risk of RRI is ‘high’ may be explained by the difference between these groups in relation to their overall duration of involvement within the sport – nearly 60% of coaches had six or more years of coaching experience, while the average training age of the distance runners was 3.4 years. These differences highlight that the coaches will have had a ‘higher level of exposure’ to running-related training and performance environments. As a result, this observed ‘higher level of exposure’ may reflect that coaches have had more opportunities to engage with resources about RRI (e.g., via coach education for qualification and/or as part of professional development), thus increasing their knowledge about RRI prevention.

Aligned to the responses of all participants about the risk of sustaining RRI, it is notable that a similar majority (88%) indicated that trying to prevent RRI is ‘very important,’ supporting the idea that seeking to prevent RRI is a priority for both distance runners and coaches. Given that previous research has highlighted that adopting injury prevention measures is a key part of a young athletes’ journey to becoming an elite senior athlete [38], this finding is encouraging. Although the reasons why participants thought preventing RRI was ‘very important’ were not explored, it should be highlighted that this can be framed positively, focused on improving the overall training availability, performance, autonomy, and longevity of competitive adolescent distance runners. Given that distance running is typically a late specialization sport [39], this positive framing (i.e., adding value) of RRI prevention measures is likely to be important for effective real-world implementation.

Survey responses related to what commonly causes RRI varied between distance runners and coaches. Of particular interest was that the distance runners identified a modifiable cause of RRI (i.e., too much training) as the most common cause of RRI, while coaches selected a non-modifiable cause of RRI (i.e., growth and maturation). One interpretation of this finding is that both groups view RRI as something that is largely beyond their control. For example, the fact that training is often prescribed by coaches allows the completion of ‘too much training’ by competitive adolescent distance runners to potentially be blamed on the coach – (re)framing this cause of RRI as non-modifiable. In turn, this raises a question about who responsibility lies with (e.g., athlete or coach) in terms of RRI prevention [40]. Growth and maturation is also a broad and complex process, and may have different interpretations in relation to how this may cause or relate to RRI [41]. Therefore, further consideration of such factors will be important as part of the larger co-creation process.

Both the distance runners and coaches who took part in this study identified that adolescent distance runners were most often exposed to lower leg and knee RRIs, with these types of RRI also being seen as the most important to prevent. In alignment with these data, lower leg and knee RRIs were the most frequently reported injury types in a previous epidemiological study involving a sample of competitive English adolescent distance runners [15]. The fact that lower leg RRIs are also seen as the most important to prevent is potentially due to the fact that bone stress injuries (e.g., medial tibial stress syndrome) are known to be common amongst adolescent distance runners [5], often more severe than other RRIs and occurring in the lower leg. As a result, it is likely that this ‘common knowledge’ influenced participant responses to this survey question.

### Current behavior

All participants (100%) reported that they either ‘did’ or ‘sometimes did’ things to prevent RRI in a normal training week. This finding is particularly promising in terms of the willingness of distance runners and coaches to engage with RRI prevention measures. Yet, self-reported weekly engagement with RRI prevention measures does not guarantee that they are being done to an appropriate standard and/or as prescribed (e.g., duration) – nor whether they are actually engaging with any preventative measures that will effectively reduce the risk of RRI. Related to compliance, results from a cluster-randomized controlled trial of an unsupervised exercise-based Athletics injury prevention program demonstrated that less than 10% of the intervention group successfully completed the program two or more times per week, as prescribed [28]. Importantly, this issue is not limited to Athletics-based injury prevention measures [42]. As a related finding, it was notable that just over half of coaches had previously received advice about implementing RRI prevention measures, compared to 90% of distance runners. This finding highlights that implementation of RRI prevention measures is likely to be guided by the distance runners, rather than being advised by their coaches – to be further considered as part of the larger co-creation process.

In terms of the types of RRI prevention measures implemented by distance runners and coaches, it was interesting to see that the majority of options presented in the survey were used at least once per week. The one exception was the use of a ‘specific prevention protocol,’ likely due to the fact that – to the best of our knowledge – such a ‘specific prevention protocol’ does not currently exist in this context (i.e., for completive adolescent distance runners). The range of currently implemented RRI prevention measures supports the idea that any subsequently created measures should include multiple different approaches, perhaps viewed as a ‘toolkit’ of options available to the athlete and/or coach.

### Need and support

The results from this study indicate that a large majority of competitive distance runners and distance running coaches view the possible creation of RRI prevention measures as ‘needed’ (i.e., an important initiative) and generally ‘feel positively’ about injury prevention measures. While this provides a high level of support for the larger co-creation process, the fact that less of a majority (∼75%) of distance runners and coaches were able to agree that they would adopt such RRI prevention measures is a minor concern. This is likely due to the fact that a number of study participants are unwilling to commit to implementation prior to seeing what is created in practice.

### Possible content and form

Given the reported need and support for RRI prevention measures, it was particularly interesting that several differences were observed between distance runners and coaches in relation to the possible content and form of such measures. Some of the key differences seem to be coupled. For example, the fact that the highest proportion of distance runners favored completion of RRI prevention measures ‘at home’ is likely related to the fact that they also preferred the idea of completing such measures ‘at a different time to training.’ In contrast, the coaches would prefer for RRI prevention measures to be completed ‘at the local athletics club’ and integrated ‘as part of a running training session.’ These differences further add to the argument surrounding who responsibility lies with for implementing RRI prevention measures and is possibly explained by both groups wanting to oversee the implementation of such measures. This finding also generates opportunities to create different potential ‘exposures’ to preventative measures, catering to both the needs of distance runners and coaches.

The observed differences between distance runners and coaches emphasize the overall complexity of developing RRI prevention measures, with several possible delivery options (see Table 4). This variety also interacts with the range of RRI measures that are currently implemented in practice by both groups (see Table 3), each used a number of different times per week. As a result, these survey results enabled elements of the subsequent workshops, as part of the larger co-creation process, to focus more precisely on what content and form is deemed to be essential within this specific population – allowing expert consensus to be reached.

### Methodological considerations

Compared to similar research [43], the sample size in this study was relatively small. However, this was restricted by the specific pool of potential participants that the sample was drawn from, selected to: (1) ensure that only those with relevant lived experience (i.e., specialist tacit knowledge) were able to take part [44] and (2) improve the likely acceptability of co-created RRI prevention measures (as part of England Athletics’ YTP), if this was viewed as an ‘important initiative.’ Despite the relatively small sample size, the overall response rate was deemed to be average-to-good. As a related methodological consideration, there is a possibility of responder bias, whereby those distance runners and coaches who already value the importance of RRI prevention may have been more likely to complete the survey.

It is important to highlight that the cross-sectional design of this study means that the presented results only provide a ‘snapshot’ of the knowledge, behavior, and needs of the distance runners and coaches enrolled on England Athletics’ YTP. This should not necessarily be viewed as a limitation but helps frame the overall importance of adopting an iterative and engaged approach to creating RRI prevention measures. Therefore, results from this study should be used to help guide the larger co-creation process, rather than simply being used to inform decisions without ensuring that the views of key stakeholders are comprehensively considered [33].

## Conclusions

The results from this study highlight key points that will help support and frame the co-creation of RRI prevention measures for competitive adolescent distance runners enrolled on England Athletics’ YTP, and affiliated distance running coaches. This includes broad agreement from both the distance runners and coaches that trying to prevent RRI is ‘very important’ and that creating RRI prevention measures is an important initiative. Alongside this support, interesting differences between the distance runners and coaches were also identified, including, for example: (1) their understanding of the most common causes of RRI and (2) their preferences about where and when to complete RRI prevention measures. Collectively, these results will help inform subsequent steps of the larger co-creation process, with an emphasis on developing multifaceted and context-specific RRI prevention measures that are deemed to be feasible and acceptable for real-world implementation.

## Supplementary Material

Supplemental Material

Supplemental Material

## Data Availability

Data are available upon reasonable request. Please contact the corresponding author to arrange access to study data.
