# Supplementary material for: Co-creation of injury prevention measures for competitive adolescent distance runners: knowledge, behavior, and needs of athletes and coaches enrolled on England Athletics’ Youth Talent Programme
Source: Ann Med. 2024 Apr 10;56(1):2334907. doi: 10.1080/07853890.2024.2334907 (PMC11008313; doi:10.1080/07853890.2024.2334907)
Supplement: Supplemental Material [file IANN_A_2334907_SM4912.docx]

**Supplementary File 2:** Copy of the online survey for distance running coaches currently affiliated to England Athletics’ Youth Talent Programme, completed via Qualtrics.

***Section #1: Background Information***

1. **Gender:** Male / Female / Prefer to self-describe / Prefer not to say.

- If “prefer to self-describe” selected, textbox provided for answer.

1. **How many years have you been actively coaching adolescent distance runners?**

- Textbox provided for answer.

1. **Please provide a description of the adolescent distance runners that you currently coach, related to the following statements (textbox provided for answers):**

- Total number of athletes in your training group.
- Performance level of athletes in this group (i.e., club, county, regional, national, or international).
- Main distance running event of the athletes in this group.
- Number of times that you would complete face-to-face training with this group per week (pre-Covid).
- Any further information.

*End of Section #1.*

***Section #2: Current Knowledge***

1. **In your opinion, how big is the risk of sustaining an injury as an adolescent distance runner?**

- High / Moderate / Low / Don’t know.

1. **As a coach, how important do you think it is for you to try to prevent injuries?**

- Very important / Important / Moderately important / Somewhat important / Not important / Don’t know.

1. **What type of injuries do you think adolescent distance runners are exposed to?**

- Hip/groin / Thigh (hamstrings or quadriceps) / Knee / Lower leg (tibia/fibula, calf, or Achilles) / Ankle / Foot / Other.
- If “other” selected, textbox provided for answer.

1. **What type of injuries do you think are most important to prevent for adolescent distance runners?**

- Hip/groin / Thigh (hamstrings or quadriceps) / Knee / Lower leg (tibia/fibula, calf, or Achilles) / Ankle / Foot / Other.
- If “other” selected, textbox provided for answer.

1. **What do you think are the most common reasons for injuries among adolescent distance runners?**

- Too little training / Too much training / Too many competitions / Not enough recovery after training sessions and/or competitions / Low muscle strength / Reduced flexibility / Poor quality running surface / Issues related to growth and maturation / Other.
- If “other” selected, textbox provided for answer.

*End of Section #2.*

***Section #3: Current Behaviour***

1. **In a normal week, do you currently implement any running-related injury prevention measures with the adolescent distance runners that you coach?**

- Yes / No / Sometimes / Don’t know.
- If “yes” or “sometimes,” what do you currently do to prevent running-related injures? And how many times per week? A matrix table was provided, including the following options:
  - **Types:** Warm-up routine / Cool-down routine / Balance and coordination training / Flexibility training / Strength training (physical preparation) / Core training (circuit training) / Stretching before running / Stretching after running / Running-specific drills / Use of taping/strapping (i.e., kinesiology tape) / use of specialised clothing, insoles, shoes, socks, etc. / Adjust different training load variables / Specific prevention protocol / Other.
  - **Times per week:** None / Once / Twice / 3x / 4x / 5x / 6x / 7x.
  - If “other” selected, textbox provided for answer.
- If “no,” textbox provided for participant to explain why not (i.e., barriers to completing injury prevention activities).

1. **Have you previously received support/advice about implementing injury prevention measures?**

- Yes / No / Don’t know.
- If “yes” selected, who have you received support/advice from? Multiple choice list provided, as follows:
  - Yourself (reading books, etc.) / Other coaches at your athletics club / England Athletics and/or UK Athletics / Your athletics coach / Other coaches that are part of a wider network / Other.
  - If “other,” textbox provided for answer.

1. **How do you feel about injury prevention measures?**
   - Very positive / Positive / Neutral / Negative / Very negative / Don’t know.

*End of Section #3.*

***Section #4: Need and Support for Injury Prevention Measures***

1. **How would you rate your current knowledge about injury prevention?**

- Excellent / Good / Average / Poor / Very poor / Don’t know.
- If “excellent” or “good,” textbox provided for participant to explain what sources of information they used to gain this knowledge.
- If “poor” or “very poor,” textbox provided for participant to explain why they think that this is the case.

1. **Do you think that the development of injury prevention measures for adolescent distance runners is an important initiative by England Athletics?**

- Very important / Important / Moderately important / Somewhat important / Not important / Don’t know.
- If “excellent” or “good,” textbox provided for participant to explain what sources of information they used to gain this knowledge.

1. **If England Athletics were to develop injury prevention measures, would you include these in your training programmes and promote them to your athletes?**

- Yes / No / Don’t know.
- If “yes,” what factors would help you to include this in your training programmes?
- If “no,” what factors would make you more likely to include this in your training programmes?

1. **At what points during the training and competition year do you think that injury prevention measures need to be a training focus?**

- During the winter training phase (including cross-country and indoor track season) / When transitioning to the outdoor track season / During the outdoor season / Following the outdoor track season (transitioning to winter training phase) / All year round (no specific point) / Other / Don’t know.
- If “other” selected, textbox provided for answer.

*End of Section #4.*

***Section #5: Content and Form of Prevention Measures***

1. **How much time per day do you think that your athletes should spend on injury prevention activities?**

- Up to 5 minutes / Between 5 and 10 minutes / Between 10 and 20 minutes / Between 20 and 30 minutes / More than 30 minutes / They should not spend any time on injury prevention activities.

1. **How many times per week do you think that your athletes should complete injury prevention activities?**

- Once per week / Twice per week / Three times per week / Four times per week / Five times or more per week / They should spend any time on injury prevention activities.

1. **What do you think are the key barriers and facilitators for ensuring that your athletes spend a suitable amount of time completing injury prevention activities?**

- Textbox provided for answer.

1. **Where do you think that your athletes would prefer to complete injury prevention activities?**

- At home / At their athletics club / At their school, academy, or college / Other / Don’t know.
- If “other” selected, textbox provided for answer.

1. **When do you think that your athletes would most like to include injury prevention activities into their training schedule?**

- As part of a running training session / At a different time to a running training session / Other / Don’t know.
- If “other” selected, textbox provided for answer.

1. **How would you like your athletes to receive information about injury prevention measures?**

- England Athletics website (i.e., Athletics Hub) / From their coach / Your athletics club website / Specific injury prevention website / Updates via email / Educational workshop/seminar / Social media (i.e., YouTube, Facebook, Instagram, etc.) / Mobile phone application / Series of videos / Infographics and posters / Other / No preference.
- If “other” selected, textbox provided for answer.

1. **Who do you think your athletes would like to deliver information about the injury prevention measures?**

- Athletics coach / Professional athlete **/** Sports physiotherapist or doctor / Strength and conditioning coach / Other / No preference.
- If “other” selected, textbox provided for answer.

1. **Is there anything else that you think would be helpful to support the health and performance of adolescent distance runners?**

- Textbox provided for answer.

*End of Section #5.*

***Section #6: Future Involvement***

1. **Would you be willing to contribute to meetings about the future direction of this project?**

- Yes / No.
- If “yes” selected, textbox provided for participant to provide email address.

1. **Please provide any other comments about this ongoing project.**

- Textbox provided for answer.

*End of Section #6.*
