# Supplementary material for: Co-creation of injury prevention measures for competitive adolescent distance runners: knowledge, behavior, and needs of athletes and coaches enrolled on England Athletics’ Youth Talent Programme
Source: Ann Med. 2024 Apr 10;56(1):2334907. doi: 10.1080/07853890.2024.2334907 (PMC11008313; doi:10.1080/07853890.2024.2334907)
Supplement: Supplemental Material [file IANN_A_2334907_SM4898.docx]

**Supplementary File 1:** Copy of the online survey for adolescent distance runners currently enrolled on England Athletics’ Youth Talent Programme, completed via Qualtrics.

***Section #1: Background Information***

1. **Date of Birth:** DD/MM/YYYY.
2. **Gender:** Male / Female / Prefer to self-describe / Prefer not to say.

- If “prefer to self-describe” selected, textbox provided for answer.

1. **Which distance running event is your main event?**

- 800m / 1,500m / 3,000m / 5,000m / 10,000m / Other.
- If “other” selected, textbox provided for answer.

1. **How many years have you taken part in distance running?**

- Textbox provided for answer.

1. **How many months of the past year (12 months) have you participated in distance running?**

- None / one / two / three / four / five / six / seven / eight / nine / ten / eleven / twelve.

1. **Is distance running more important to you than any other sport?**

- Yes / No / Don’t know.

1. **Have you quit other sports in order to focus on distance running?**

- Yes / No / Don’t know.

*End of Section #1.*

***Section #2: Current Knowledge***

1. **In your opinion, how big is the risk to you as a distance runner of sustaining an injury?**

- High / Moderate / Low / Don’t know.

1. **As a distance runner, how important do you think it is for you to try to prevent injuries?**

- Very important / Important / Moderately important / Somewhat important / Not important / Don’t know.

1. **As a distance runner, what type of injuries do you think you are exposed to?**

- Hip/groin / Thigh (hamstrings or quadriceps) / Knee / Lower leg (tibia/fibula, calf, or Achilles) / Ankle / Foot / Other.
- If “other” selected, textbox provided for answer.

1. **As a distance runner, what type of injuries do you think are most important to prevent?**

- Hip/groin / Thigh (hamstrings or quadriceps) / Knee / Lower leg (tibia/fibula, calf, or Achilles) / Ankle / Foot / Other.
- If “other” selected, textbox provided for answer.

1. **What do you think are the most common reasons for injuries among adolescent distance runners?**

- Too little training / Too much training / Too many competitions / Not enough recovery after training sessions and/or competitions / Low muscle strength / Reduced flexibility / Poor quality running surface / Issues related to growth and maturation / Other.
- If “other” selected, textbox provided for answer.

*End of Section #2.*

***Section #3: Current Behaviour***

1. **In a normal week, do you currently do anything to try and prevent running-related injures?**

- Yes / No / Sometimes / Don’t know.
- If “yes” or “sometimes,” what do you currently do to prevent running-related injures? And how many times per week? A matrix table was provided, including the following options:
  - **Types:** Warm-up routine / Cool-down routine / Balance and coordination training / Flexibility training / Strength training (physical preparation) / Core training (circuit training) / Stretching before running / Stretching after running / Running-specific drills / Use of taping/strapping (i.e., kinesiology tape) / use of specialised clothing, insoles, shoes, socks, etc. / Adjust different training load variables / Specific prevention protocol / Other.
  - **Times per week:** None / Once / Twice / 3x / 4x / 5x / 6x / 7x.
  - If “other” selected, textbox provided for answer.
- If “no,” textbox provided for participant to explain why not (i.e., barriers to completing injury prevention activities).

1. **Have you previously received support/advice about injury prevention?**

- Yes / No / Don’t know.
- If “yes” selected, who have you received support/advice from? Multiple choice list provided, as follows:
  - Yourself (reading books, etc.) / Your parents/carers / Your athletics coach / Other athletes in your training group / Your athletics club / Your school, academy, or college / England Athletics and/or UK Athletics / Other.
  - If “other,” textbox provided for answer.

1. **How do you feel about injury prevention measures?**
   - Very positive / Positive / Neutral / Negative / Very negative / Don’t know.

*End of Section #3.*

***Section #4: Need and Support for Injury Prevention Measures***

1. **How would you rate your current knowledge about injury prevention?**

- Excellent / Good / Average / Poor / Very poor / Don’t know.
- If “excellent” or “good,” textbox provided for participant to explain what sources of information they used to gain this knowledge.
- If “poor” or “very poor,” textbox provided for participant to explain why they think that this is the case.

1. **Do you think that the development of injury prevention measures for adolescent distance runners is an important initiative by England Athletics?**

- Very important / Important / Moderately important / Somewhat important / Not important / Don’t know.
- If “excellent” or “good,” textbox provided for participant to explain what sources of information they used to gain this knowledge.

1. **If England Athletics were to develop injury prevention measures, would you include these as part of your training?**

- Yes / No / Don’t know.
- If “yes,” what factors would help you to include this in your training?
- If “no,” what factors would make you more likely to include this in your training?

1. **At what points during the training and competition year do you think that injury prevention measures need to be a training focus?**

- During the winter training phase (including cross-country and indoor track season) / When transitioning to the outdoor track season / During the outdoor season / Following the outdoor track season (transitioning to winter training phase) / All year round (no specific point) / Other / Don’t know.
- If “other” selected, textbox provided for answer.

*End of Section #4.*

***Section #5: Content and Form of Prevention Measures***

1. **How much time per day would you be willing to spend on injury prevention activities?**

- Up to 5 minutes / Between 5 and 10 minutes / Between 10 and 20 minutes / Between 20 and 30 minutes / More than 30 minutes / I would not spend any time on injury prevention activities.

1. **How many times per week would you be willing to spend on injury prevention activities?**

- Once per week / Twice per week / Three times per week / Four times per week / Five times or more per week / I would not spend any time on injury prevention activities.

1. **Where would you prefer to complete these injury prevention activities?**

- At home / At your athletics club / At your school, academy, or college / Other / Don’t know.
- If “other” selected, textbox provided for answer.

1. **When would you most like to include injury prevention activities into your training schedule?**

- As part of a running training session / At a different time to a running training session / Other / Don’t know.
- If “other” selected, textbox provided for answer.

1. **How would you like to receive information about injury prevention measures?**

- England Athletics website (i.e., Athletics Hub) / From your coach / Your athletics club website / Specific injury prevention website / Updates via email / Educational workshop/seminar / Social media (i.e., YouTube, Facebook, Instagram, etc.) / Mobile phone application / Series of videos / Infographics and posters / Other / No preference.
- If “other” selected, textbox provided for answer.

1. **Who would you like to deliver information about the injury prevention measures?**

- Athletics coach / Professional athlete **/** Sports physiotherapist or doctor / Strength and conditioning coach / Other / No preference.
- If “other” selected, textbox provided for answer.

1. **Would you also like to receive advice that supports adolescent distance runners who have an injury and/or are recovering from an injury?**

- Yes / No / Don’t know.
- If “yes,” textbox provided for participant to explain what advice they would like to receive.

1. **Is there anything else that you think would be helpful to support the health and performance of adolescent distance runners?**

- Textbox provided for answer.

*End of Section #5.*

***Section #6: Future Involvement***

1. **Would you be willing to contribute to meetings about the future direction of this project?**

- Yes / No.
- If “yes” selected, textbox provided for participant to provide email address.

1. **Would your parents/carers be willing to contribute to meetings about the future direction of this project?**

- Yes / No.
- If “yes” selected, textbox provided for participant to add contact information.

1. **Please provide any other comments about this ongoing project.**

- Textbox provided for answer.

*End of Section #6.*
